# *TP53* mutations detected in circulating tumor cells present in the blood of metastatic triple negative breast cancer patients

**DOI:** 10.1186/s13058-014-0445-3

**Published:** 2014-10-09

**Authors:** Sandra V Fernandez, Catherine Bingham, Patricia Fittipaldi, Laura Austin, Juan Palazzo, Gary Palmer, Katherine Alpaugh, Massimo Cristofanilli

**Affiliations:** 1 Department of Medical Oncology, Thomas Jefferson University, Philadelphia, 19107 PA USA; 2 Fox Chase Cancer Center, Philadelphia, 19111 PA USA; 3 Department of Pathology, Thomas Jefferson University, Philadelphia, 19107 PA USA; 4 Foundation Medicine, Cambridge, 02141 MA USA

## Abstract

**Introduction:**

Circulating tumor cells (CTCs) are tumor cells shed from either primary tumors or its metastases that circulate in the peripheral blood of patients with metastatic cancers. The molecular characterization of the CTCs is critical to identifying the key drivers of cancer metastasis and devising therapeutic approaches. However, the molecular characterization of CTCs is difficult to achieve because their isolation is a major technological challenge.

**Methods:**

CTCs from two triple negative breast cancer patients were enriched using CellSearch and single cells selected by DEPArray™. A *TP53* R110 fs*13 mutation identified by next generation sequencing in the breast and chest skin biopsies of both patients was studied in single CTCs.

**Results:**

From 6 single CTC isolated from one patient, 1 CTC had *TP53* R110 delC, 1 CTC showed the *TP53* R110 delG mutation, and the remaining 4 single CTCs showed the wild type p53 sequence; a pool of 14 CTCs isolated from the same patient also showed *TP53* R110 delC mutation. In the tumor breast tissue of this patient, only the *TP53* R110 delG mutation was detected. In the second patient a *TP53* R110 delC mutation was detected in the chest wall skin biopsy; from the peripheral blood of this patient, 5 single CTC and 6 clusters of 2 to 6 CTCs were isolated; 3 of the 5 single CTCs showed the *TP53* R110 delC mutation and 2 CTCs showed the wild type *TP53* allele; from the clusters, 5 showed the *TP53* R110 delC mutation, and 1 cluster the wild type *TP53* allele. Single white blood cells isolated as controls from both patients only showed the wild type *TP53* allele.

**Conclusions:**

We are able to isolate uncontaminated CTCs and achieve single cell molecular analysis. Our studies showed the presence of different CTC sub-clones in patients with metastatic breast cancer. Some CTCs had the same TP53 mutation as their matching tumor samples although others showed either a different TP53 mutation or the wild type allele. Our results indicate that CTCs could represent a non-invasive source of cancer cells from which to determine genetic markers of the disease progression and potential therapeutic targets.

**Electronic supplementary material:**

The online version of this article (doi:10.1186/s13058-014-0445-3) contains supplementary material, which is available to authorized users.

## Introduction

Molecular characterization of primary tumors has greatly contributed to the personalized treatment of breast cancer patients [1]-[6]. Unfortunately, there is still a population of patients that develop recurrence in spite of adequate multidisciplinary treatment of their primary tumor and ultimately succumb to metastatic disease [7]. It has been shown that metastases, which may develop several years after occurrence of the primary tumor, can differ greatly from primary tumor tissue in terms of genetic characteristics [8]-[12]. Although the molecular characterization of metastases will improve the currently available prognostic and predictive models, taking biopsies from metastases in patients is an invasive procedure that is frequently impossible due to the lack of accessible lesions. Circulating tumor cells (CTC) isolated from the blood of patients with metastatic carcinoma provide a source of tumor cells and can be a potential replacement for such repeatable tumor biopsies.

CTC are tumor cells shed from either the primary tumor or its metastases that circulate in the peripheral blood of patients and can thus be regarded as liquid biopsies of metastasizing cells. Little is known about the timing of CTC release from primary tumors, their heterogeneity, or their functional properties. Although their exact composition is unknown, a fraction of these cells are thought to be viable metastatic precursors capable of initiating a clonal metastatic lesion [13]. CTCs have been detected in a majority of epithelial cancers, including those from prostate [14], colorectal [15], and breast cancers [16]. The molecular characterization of CTCs is important because it may enable insight into the molecular biology of metastasis, and the association of their molecular profiles with treatment outcomes, and reveal the presence of potential therapeutic targets.

Triple-negative breast cancer (TNBC), which is defined by the absence of estrogen and progesterone receptors, and a lack of human epidermal growth factor receptor-2 (HER2) overexpression, has a poor prognosis and molecular profiling generally reveals a basal-like subtype. It was found that 83% of basal-like tumors had TP53 mutations compared to 15% in the luminal, normal-like and HER2-positive groups combined [17]. Given the high frequency of *TP53* mutations in hormone-receptor-negative tumors and their association with poor prognostic features, TP53 seems to play a pivotal role in tumor progression in TNBC [18]. *TP53* is a tumor suppressor gene which encodes a 393-amino-acid nuclear phospho-protein that prevents propagation of genetically altered cells [19]. It was found that suppressing TP53 function in squamous cell carcinoma (SCC) cells reduced anoikis in suspension cultures [20]. Mutant TP53 plays a role in the epithelia-mesenchymal transition by inhibiting epithelial markers such as E-cadherin [21] and promoting transcription of genes associated with a mesenchymal phenotype such as Twist, ZEB-1 and ZEB-2 [22],[23]. These processes facilitate the creation of cancer stem-like cells which promote tumor growth and metastatic spread [24]. TP53 expression has a high correlation with Ki67 expression and is associated with higher histologic grade, larger tumor size, and co-expression of vascular endothelial growth factor (VEGF), epidermal growth factor receptor (EGFR) and Type II topoisomerase (TOPO II) [25].

The objective of this work was to genetically characterize CTCs isolated from metastatic TNBC patients by examining a *TP53* mutation previously found in the corresponding patient’s tissue tumor samples.

## Methods

### Patients

Patients with metastatic inflammatory breast cancer (IBC) undergoing systemic treatment for their conditions were evaluated before starting a new treatment. Patients signed informed consent and HIPAA certification from the Human Subject Protection Committee prior to sample collection. This study was approved by both the Research Review Committee (RRC) and Institutional Review Board (IRB) at Fox Chase Cancer Center. The TRF001321 patient was diagnosed with triple-negative IBC in her right breast and invasive ductal carcinoma non-IBC in her left breast in 2011; the patient had a double mastectomy in October 2011 and a breast tumor sample from her right breast was sent for sequencing. The TRF000155 patient was diagnosed with triple-negative IBC in 2008; chest wall skin biopsy was obtained in 2012 and sent for sequencing. Blood from both patients was drawn in 2012 for CTCs studies. Both patients passed away in 2012.

### Tumor tissue sample sequencing

Formalin-fixed paraffin embedded (FFPE) tumor tissues (primary breast biopsy/mastectomy and/or chest wall skin 4- to 6-mm punch biopsies) were prepared: 10 (5 to 10 μm) unstained sections were cut and placed on charged slides and submitted to Foundation Medicine (Cambridge, MA, USA) for genetic analysis. DNA was isolated from the fixed cells and genomic analysis was performed using next-generation sequencing (NGS) (Foundation One™). Foundation One™ is a comprehensive, NGS-based, cancer gene test which is routinely applied to FFPE clinical samples. The assay sequences the entire coding sequence of 182 cancer-related genes (3,230 exons) plus 37 introns from 14 genes often rearranged in cancer. Briefly, hybridization capture of 3,230 exons from 182 cancer-related genes and 37 introns of 14 genes commonly rearranged in cancer was applied to ≥50 ng of DNA extracted from FFPE tumor specimens and sequenced to a unique median depth of >800×. Reads were mapped to human genome reference and alignment results were analyzed by customized tools.

### Tumor tissue immunohistochemistry

Immunohistochemical staining was performed on 5-μm FFPE sections. The monoclonal mouse anti-human p53 clone DO-7 (Dako, Catalog number M7001) was used according to the manufacturer’s instructions. Briefly, after deparaffinization and rehydration, sections were subjected to heat-induced epitope retrieval by steaming in 0.01 M citrate buffer (pH 6.0) for 20 minutes. After endogenous peroxidase activity was quenched with 3% hydrogen peroxide for 20 minutes and nonspecific protein binding was blocked with goat serum, sections were incubated overnight with primary antibodies to TP53 (Dako, DO-7, mouse, 1:200) at 4°C, followed by biotinylated goat anti-mouse IgG (LSAB kit, Dako) for 30 minutes. The sections were rinsed in PBS and then incubated with horseradish peroxidase-labeled streptavidin-biotin complex for 30 minutes. The sections were then visualized with the chromogen 3, 3′-diaminobenzidine (DAB) containing 0.05% hydrogen peroxide and then lightly counterstained with Mayer’ hematoxylin. As a negative control, the primary antibody was replaced with mouse non-immunized IgG. A positive TP53 ovarian tumor was used as control.

### Blood samples

**Figure 1:**
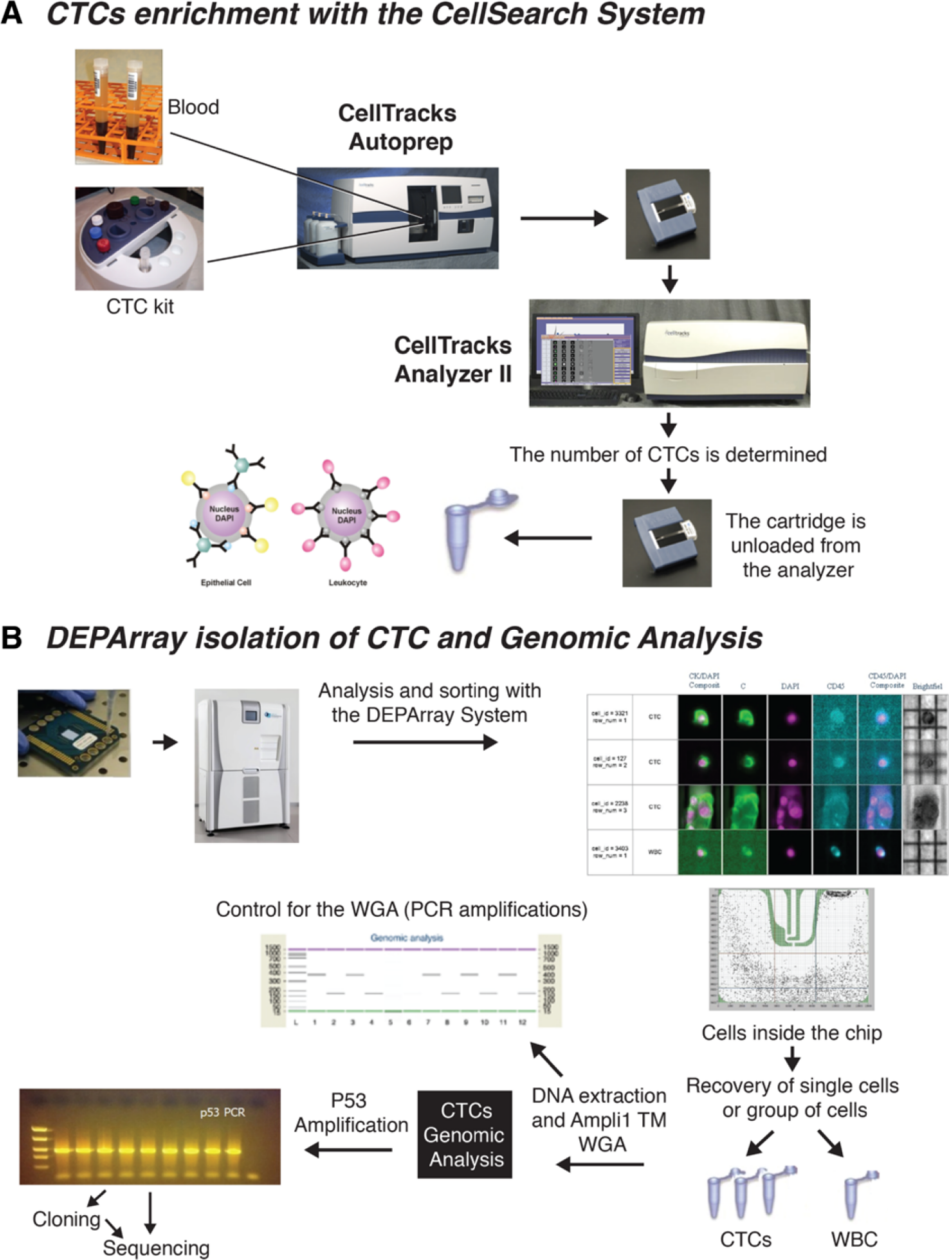
**Circulating tumor cells (CTCs) were isolated from the blood of metastatic Triple-negative breast cancer (TNBC) patients using the CellSearch** **™** **followed by the DEPArray** **™** **system.** **(A)** CTC were enriched using the CellSearch CTC kit (Veridex) which uses anti-EpCAM conjugated ferrofluid, anti-pan CK-phycoerythrin (PE), anti-CD45-allophyocyanin (APC) and 4′, 6 diamidino 2 phenylindole for nuclear staining. **(B)** Single CTCs, clusters of CTCs and single white blood cells (WBCs; used as controls) were selected and isolated on the DEPArray™ System. Two single CTCs and a big CTC cluster selected from a triple-negative patient (TRF106811/6146) are shown. In the upper part of the figure, the anti- EpCAM and anti-CK antibodies are represented by blue and yellow circles, respectively, around a epithelial cell; the anti-CD45 antibodies are represented by pink circles around a leukocyte.

One tube of 7.5 ml blood from the patients with metastatic breast cancer was drawn into CellSave Preservative tubes (Veridex, LLC) for CTC enrichment and enumeration. The schematic representation of the procedure that was followed to isolate a single CTC is shown in Figure 1. The CellSearch™ System was used to determine if CTCs were present in the patients’ blood and the resulting sample served as a primary enrichment of CTC that was used for single-cell selection using the DEPArray™ System (Silicon Biosystems, San Diego, CA, USA).

### Immuno-magnetic enrichment and enumeration of CTCs

Standard protocols and reagents for CTC enrichment and enumeration using the Food and Drug Administration (FDA)-approved CellSearch™ system (Veridex LLC) - CellTracks Autoprep and CellTracks Analyzer II - were employed. Blood samples were collected as indicated before and processed according to the manufacturer’s instructions using the CellSearch CTC kit (Veridex LLC). Briefly, CTCs were enriched on the CellTracks Autoprep using ferrofluid conjugated with anti-epithelial cell adhesion molecule (EpCAM) antibody. Captured cells were stained with fluorescently labeled monoclonal antibodies specific for pan cytokeratin (CK-8/18/19-phycoerythrin (PE)), leukocyte common antigen (CD45-allophyocyanin (APC)), and nuclear-stained (with (4′, 6 diamidino 2 phenylindole (DAPI)). Captured cells were automatically loaded by the system into a cartridge held within a MagNest magnetic holder which was then placed in the CellTracks Analyzer II. Serial pictures were taken and frames were analyzed using computer-assisted software to determine if the events expressed cytokeratin-phycoerythrin (PE) and DAPI staining. Tumor cells were defined using standard CellSearch CTC criteria, these being: round to oval shape, presence of a clear DAPI-stained nucleus, at least 50% overlap between the CK-PE-positive cytoplasm and the nucleus, and CD-45-APC negative. Patient samples containing a minimum of 30 CTCs were prepared for single-cell selection using DEPArray™ (Silicon Biosystems, San Diego, CA, USA).

### Sample transfer from CellSearch to DEPArray™ cartridges

Cells were removed from the cartridges and transferred into a Protein LoBind tube (Eppendorf) and the cartridge was washed twice with elution buffer and all samples combined. The samples were centrifuged at 600 × g for 5 minutes and the supernatant was carefully removed. The cell pellets were stored and protected from light at 4°C in a residual volume of elution buffer until use. This procedure was performed on the same day the sample was run on the CellTracks, as delaying removal of cells from the cartridge decreased the number of CTCs recovered.

### Isolation of single CTCs

After the CellSearch enrichment, the CTCs were selected and isolated using the DEPArray™ (Silicon Biosystems) (Figure 1B). The system is an automated platform that uses dielectrophoresis and a high-quality image-based cell selection system that allows for the identification and recovery of individual cells from heterogeneous samples. The DEPArray™ chip consists of various microelectrodes that create electric cages into which individual cells are trapped and, by alternatively activating and deactivating the microelectrodes, the cells are moved to a position in the chip that allows their recovery. Briefly, DEPArray™ cartridges (DEPArray™ A300K-cartridge, Silicon Biosystems) were loaded with 800 μl of SB115 buffer and 14 μl of sample, placing approximtely 9.26 μl of sample within the electrophoretic chamber. Upon the application of a preprogrammed electric field, cells moved to and were held within their nearest electrically controlled cages. The cartridges allowed for either 16,000 or 40,000 cells to be trapped. Images of each cage were captured with white light exposure and each of three fluorescent filter cubes (PE, APC, and DAPI/Hoechst). Cells were automatically detected by the system based on a DAPI/ Hoechst fluorescence threshold and assigned a unique cell ID. Captured images were digitally processed using multiple parameters outlined by the operator and presented in a software module that enables selection of cells of interest.

Next, in the recovery step, selected cells were electrically moved to a parking area adjacent to the main microchamber in the cartridge (Figure 1). Finally, individual cells were moved from the parking area for recovery and flushed from the chamber with three drops of SB115 buffer (30 to 40 μl) into a 200-μl PCR tube. The entire cell-routing process was monitored under bright field imaging. Routing and paths were automatically calculated by the software and routing parameters, such as speed, can be manually adjusted by the operator. A cell pellet was prepared as recommended by the supplier. Briefly, the tubes were spun at 14,000 × g for 10 minutes, 100 μl PBS was added and the sample was centrifuged again for 25 minutes. The buffer was removed from the tube and the cell pellets were stored at −80°C until further use for whole genome amplification (WGA). Individual CTCs or clusters, classified as α-cytokeratin (PE)-positive, CD45 (APC)-negative and DAPI-positive cells, were recovered in several tubes for genomic analysis. Also, individual white blood cells (WBCs) classified as CD45 (APC)-positive, CK (PE)-negative, and DAPI-positive cells, were selected and recovered as single cells to use as controls in the genomic studies.

### Whole genome amplification (WGA)

To allow genotyping analysis of single CTCs, WGA was performed using the Ampli1™ WGA Kit (Silicon Biosystems). The Ampli1™ WGA kit uses a polymerase with proofreading activity with a lower error rate (4.8 × 10^−6^) with respect to standard Taq DNA polymerases. The isolated CTCs were thawed on ice and brought up to a volume of 1 μl for the WGA procedure; single WBCs were also subjected to WGA in order to use as controls for TP53 analysis. Global amplification consisting of DNA isolation, restriction digestion, adaptor ligation and PCR amplification were performed as recommended by the supplier. Briefly, cells were lysed overnight and then digested with *MseI* restriction enzyme; adapters were ligated onto the digested DNA and fragments were then amplified by PCR with time and temperature gradients using adapter specific primers; the final volume of the sample after amplification was 50 μl. As control for the WGA, products were subjected to an end-point PCR for two control genomic DNA sequences of 373 and 167 bp (Figure 1B), respectively (Ampli1™ QC kit; SB); 2 μl of the Amli1™ WGA product was used as template per reaction and PCR products were analyzed by gel electrophoresis on the Agilent 2100 Bioanalyzer using the DNA 1000 kit (Agilent Technologies, Santa Clara, CA, USA). Only samples that showed both bands were used to study *TP53* exon 4.

### TP53 exon 4 amplification

The primers, reverse 5′ AGGCATTGAAGTCTCAGGAAG 3′ and, forward 5′CAATGGATGATTTGATGCTGTC 3′, were used to amplify a 304-bp region of *TP53* exon 4 in the CTCs. The same reverse primer (5′ AGGCATTGAAGTCTCAGGAAG 3′) and the forward primer, 5′ CCTTCCCAGAAAACCTACCAG 3′, were also used to amplify a 128-bp of this TP53 region and confirmed the sequences. PCR reactions were performed using 2 μl of the Amli1™ WGA product, GoTaq (Promega), 2 μM primers, and 52°C as the annealing temperature. The PCR products were cleaned using the QIAquick PCR purification kit and sequenced using the ABI 3130XL capillary genetic analyzer. *TP53* exon 4 was also amplified using as templates the WGA products obtained from single WBCs; the amplified region from WBCs were used as controls of *TP53* wild-type allele. PCR products were sequenced in the ABI 3130XL capillary genetic analyzer and sequences were analyzed using the Sequencher software.

### Cloning

PCR products obtained from a pool of cells were cloned using TOPO-TA Cloning Kit for Sequencing (Life Technologies) according to the manufacturer's instructions. The product of the ligation reaction was transformed into *Escherichia coli* DH5α, plated on Luria Bertani agar plates and selected with 100 μg/ml of ampicillin. Ten colonies were picked and grown in Luria Bertani media in the presence of ampicillin. Plasmid extractions were done using the Wizard Plus SV Miniprep Purification Kits (Promega) and the inserts were sequenced in the ABI 3130XL capillary genetic analyzer and sequences were analyzed using the Sequencher software as described before.

## Results

### Mutation analysis of tissue samples from metastatic breast cancer patients

**Figure 2:**
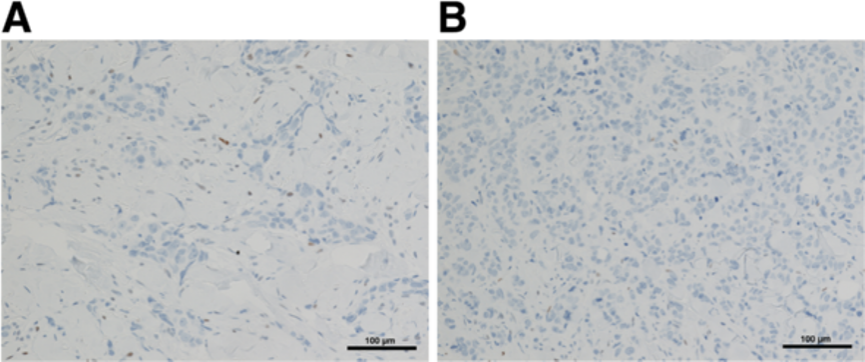
**TP53 immunohistochemical staining in the biopsies of patients with metastatic triple-negative breast cancer.** The invasive ductal carcinoma in inflammatory breast cancer (IBC) patients involving the dermis of the skin is shown. **(A)** Patient ID: TRF001321; **(B)** patient ID: TRF000155. TP53 immunohistochemical stains are negative for both. Tumor cells are present also in lymphatic spaces. Magnification: × 150.

Two patients with metastatic triple-negative (estrogen receptor (ER)-negative, progesterone receptor (PgR)-negative, HER2/neu-negative) inflammatory breast cancer were included in this study. NGS-based cancer gene test (Foundation One™) was used to detect genomic alterations in breast tumor and skin biopsies of the patients. The *TP53* R110 fs*13 mutation was detected in both breast and chest wall skin biopsies of both IBC patients. *TP53* R110 fs*13 is a frame shift deleterious mutation that produced a stop codon at position 122 (nucleotide 366) of *TP53* with the loss of the protein. In patient TRF001321, a G deletion was detected (nucleotide 329, delG) in the breast tumor sample and, in TRF000155 a C deletion (nucleotide 328, delC) was detected in the chest wall skin biopsy by NGS. The invasive ductal carcinomas present in the dermis and lymphatic system from both patients were negative for TP53 immunohistochemical stains (Figure 2); scattered cells in the epidermis were positive for TP53 (not shown). Patient TRF000155 also had mutations in *BRCA2* (*BRCA2* A1327 fs*4) and *RB1* (*RB1* F720 > *) and amplification of MYC and CCNE1 (cyclin E1) by NGS of her tissue biopsies. The sample of patient TRF001321 did not show mutations other than in *TP53*; however, it showed amplification of MYC, *MCL1* (myeloid cell leukemia sequence 1) and *JUN*.

### Circulating tumor cell (CTC) in patients with triple-negative metastatic breast cancer

**Figure 3:**
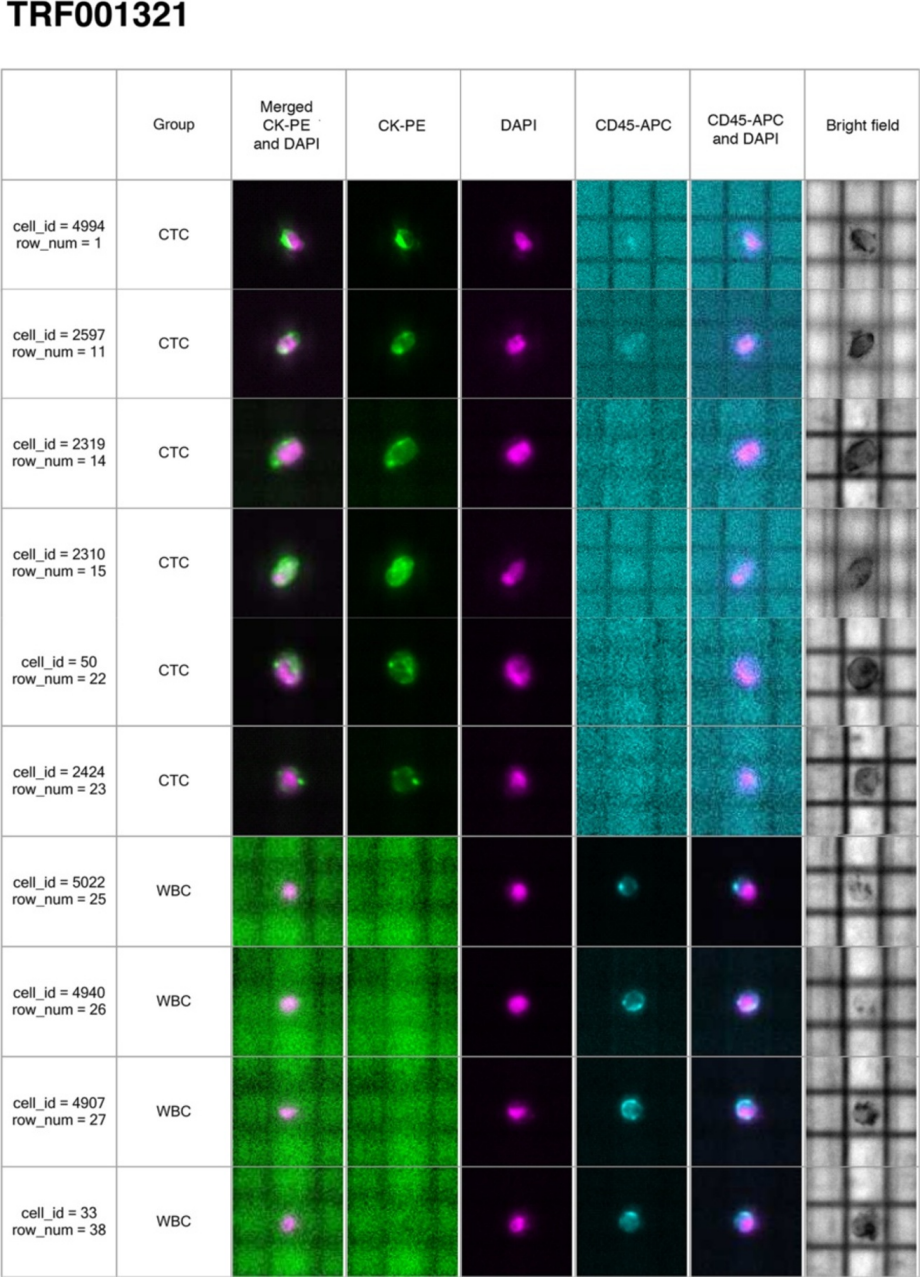
**Single circulating tumor cells (CTCs) from a patient (TRF001321) with triple-negative metastatic breast cancer visualized in the DEPArray** **™** **.** Tumor cells were defined as presence of a clear 4', 6 diamidino 2 phenylindole (DAPI)-stained nucleus, CK-phycoerythrin (PE)-positive cytoplasm and CD-45-APC negativity. Separate images for PE (green), DAPI (magenta) and APC (blue) fluorescence and bright field channels and merged CK-PE/DAPI and CD45-APC/DAPI images are shown. Six single CTCs from a metastatic triple-negative inflammatory breast cancer patient are shown; also four white blood cells (WBC) collected to use as controls for the molecular studies are shown.

**Figure 4:**
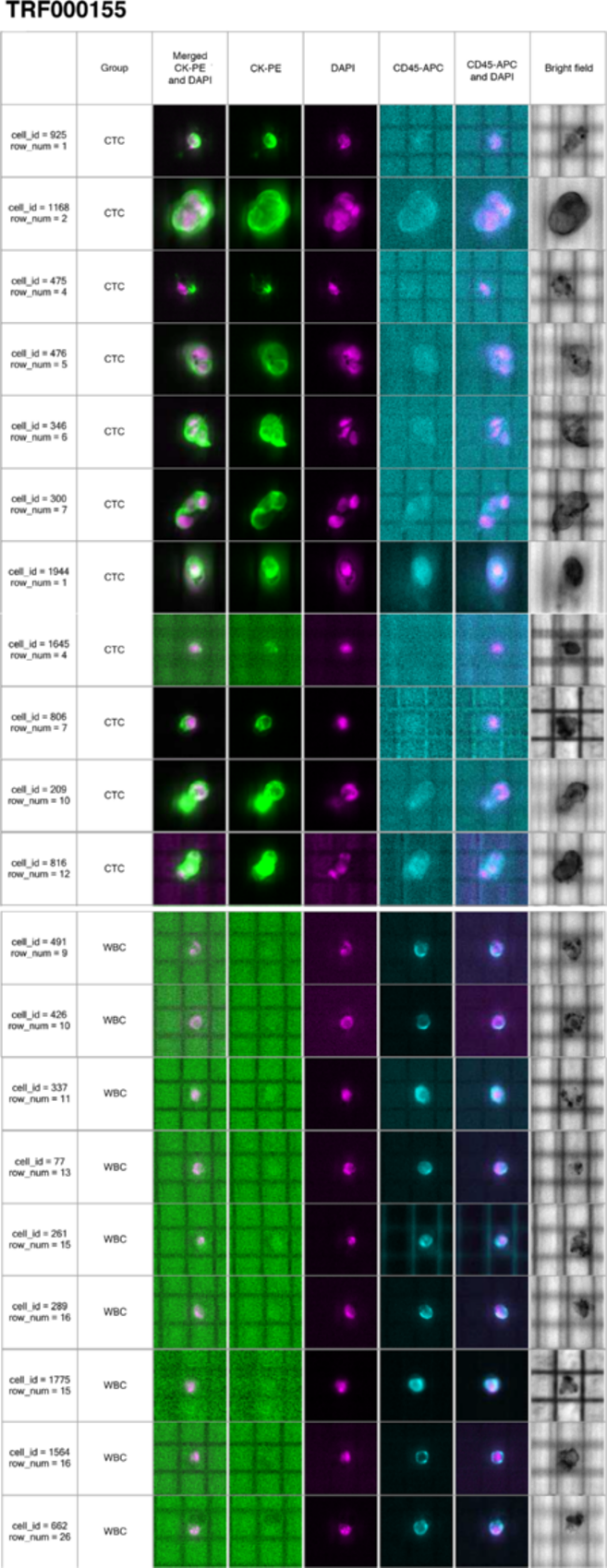
**Single circulating tumor cells (CTCs) and clusters of CTCs from the triple-negative metastatic patient TRF000155 visualized in the DEPArray** **™** **system.** Single CTCs are indicated with cell id numbers: 925, 475, 1944, 1645 and 806; clusters of CTCs are indicated with the cell id numbers: 1168, 476, 346, 300, 209 and 816. Nine white blood cells (WBC) are also shown.

CTCs were isolated from the blood of the same patients in which mutations in the breast tissue biopsies were studied. The total number of CTCs was high for both patients: patient TRF001321 had 222 CTC/7.5 ml of blood and patient TRF000155 had 45 CTC/7.5 ml of blood. The CTCs from patient TRF001321 were seen as individual cells when visualized in the DEPArray system (Figure 3); six single CTC and a pool of fourteen CTCs were recovered from this patient. The second IBC patient, TRF000155, had a mixed population of single CTCs and clusters of two to six CTCs (Figure 4); from this patient, five single CTC and six clusters were recovered for molecular analysis. Single WBCs were also isolated from both patients to use as controls in the molecular studies of *TP53*; four single WBCs were isolated from patient TRF001321 (WBC in Figure 3) and nine single WBCs were isolated from patient TRF000155 (WBC in Figure 4).

### Mutation analysis of TP53 in CTCs

**Figure 5:**
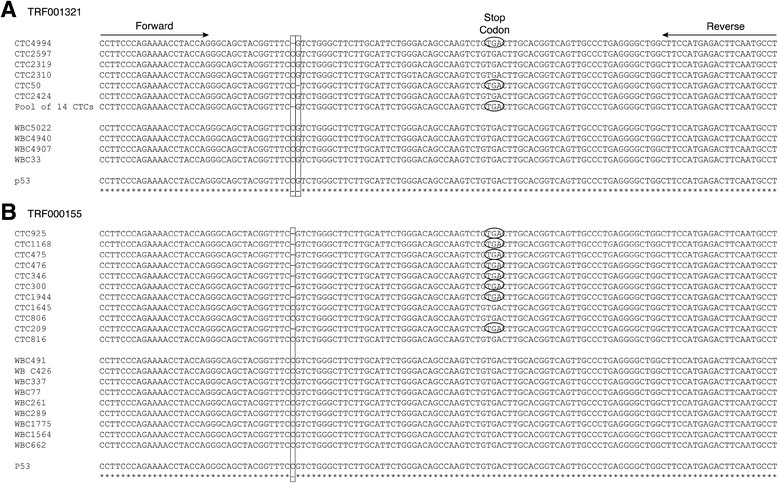
***TP53*** **exon 4 sequences in CTCs isolated from the triple-negative metastatic patients.** **(A)** Patient ID: TRF001321; **(B)** patient ID: TRF000155. The *TP53* mutations and wild- type sequences present in the selected CTCs are shown; the TP53 exon 4 sequences are also shown for the isolated white blood cells (WBC). The TP53 R110 fs*13 mutations (delC in nucleotide 328 or, delG in nucleotide 329) renders a nonfunctional TP53 by creating a premature stop codon at position 122 of the protein (nucleotide 366). The stop codons generated by the *TP53* mutation in some CTCs are shown by an encircle TGA sequence. The mutated regions are indicated in the sequences by vertical boxes.

A total of four WBCs as control, six single CTCs and a pool of fourteen CTCs were isolated from patient TRF001321 (Figure 3). The region of *TP53* exon 4 that was found mutated in the matching tissue samples was studied in these cells. In patient TRF001321, from six single CTCs isolated, one exhibited the same *TP53* R110 delG mutation (Figure 5: cell_id = 50) found in the breast tumor tissue and one showed a *TP53* R110 delC mutation (Figure 5: cell_id = 4994); the remaining four single CTCs contained the wild-type *TP53* sequence (Figure 5: cell_id = 2597, 2319, 2310, 2424). The same region of *TP53* exon 4 was amplified from a pool of 14 CTCs and the PCR product was cloned into the pCR4-TOPO vector (Life Science); 10 independent clones were selected for sequencing and all showed the *TP53* R110 delC mutation. The four WBCs (Figure 5, cell_id = 5022, 4940, 4907, 33) studied from the same patient showed the wild-type *TP53* allele, indicating lack of mutation in the germline DNA.

From patient TRF000155, who had single CTCs and clusters of CTCs, three of the five single CTCs (Figure 5: cell_id = 925, 475, 1944) revealed the *TP53* R110 delC mutation while the remaining two single CTCs had the wild-type allele (Figure 5: cell_id: 1645, 806). Five of the six clusters showed the *TP53* R110 delC mutation (Figure 5: cell_id: 1168, 476, 346, 300, 209) and one cluster had the wild-type allele (Figure 5: cell_id: 816); all nine WBCs isolated from this patient had the wild-type *TP53* allele (Figure 5: cell_id: 491, 426, 337, 77, 261, 289, 1775, 1564, 662). Table 1 summarizes all the CTCs selected and a description of the *TP53* mutations exhibited.

**Table 1:** **Circulating tumor cells (CTCs) harboring the TP53 exon 4 mutation and wild-type allele**

Patient ID	TP53 mutation	
	Isolated CTCs	Isolated white blood cells (WBCs)
TRF001321	Single CTC (4994): TP53 R110 delC fs*13	Single WBC (5022): no mutation
	Single CTC (2597): no mutation	Single WBC (4940): no mutation
	Single CTC (2319): no mutation	Single WBC (4907): no mutation
	Single CTC (2310): no mutation	Single WBC (33): no mutation
	Single CTC (50): TP53 R110 delG fs*13	
	Single CTC (2424): no mutation	
	Pool of 14 CTCs: TP53 R110 delC fs*13	
TRF000155	Single CTC (925): TP53 R110 delC fs*13	Single WBC (491): no mutation
	Cluster of 6 CTCs (1168): TP53 R110 delC fs*13	Single WBC (426): no mutation
	Single CTC (475): TP53 R110 delCfs*13	Single WBC (337): no mutation
	Cluster of 2 CTCs (476): TP53 R110 delC fs*13	Single WBC (77): no mutation
	Cluster of 3 CTCs (346): TP53 R110 delC fs*13	Single WBC (261): no mutation
	Cluster of 3 CTCs (300): TP53 R110 delC fs*13	Single WBC (289): no mutation
	Single CTC (1944): TP53 R110 delC fs*13	Single WBC (1775): no mutation
	Single CTC (1645): no mutation	Single WBC (1564): no mutation
	Single CTC (806): no mutation	Single WBC (662): no mutation
	Cluster of 2 CTCs (209): TP53 R110 delC fs*13	
	Cluster of 3 CTCs (816): no mutation	

CTCs and WBCs were isolated from two patients with metastatic triple-negative breast cancer. The cells shown in Figures 3 and 4 were studied; cell ids are indicated (in parenthesis). As control for the wild-type TP53 allele, four, and nine, WBCs from patients TRF001321 and TRF000155, respectively, were isolated and TP53 were sequenced; del, deletion; fs*13, frameshift mutation that creates a premature stop codon 13 aminoacids downstream.

## Discussion

CTCs were enriched from two patients with metastatic TNBC using the CellSearch™ method followed by single-cell selection using the DEPArray™ system. Both patients revealed high numbers of CTCs in the peripheral blood; in one patient, CTCs were seen as individual cells, and the second patient showed a mixed population of single cells and clusters of CTCs (also known as circulating tumor microemboli or CTM). Genomic analysis of the isolated CTCs revealed a heterogeneous population where some CTCs showed the same *TP53* mutation as detected in the breast and chest wall skin tumor biopsies; meanwhile, other CTCs showed a different TP53 mutation, and some the wild-type allele. As control, several single WBCs from both patients were also isolated and the genomic analysis of their amplified DNA showed only the wild-type TP53 in all of them, indicating 100% purity of the sorted cells.

In this work, we focused our studies on *TP53*. The *TP53* R110 fs*13 mutation found in both IBC patients included in this study is a deleterious mutation that renders a nonfunctional TP53 by creating a premature stop codon at position 122*.* These mutations are located in the TP53 domain for binding to the DNA where most of p53 mutations in cancer have been described [26]. A non-functional p53 pathway offers survival advantages to the cell, facilitating cell growth and anoikis resistance and the emergence of potentially more aggressive malignancy. *TP53* mutations are exceptionally frequent in cancer and are among the key driving factors in TNBC [18]. In a recent study in which NGS had been performed on tumor tissues of TNBC patients, seven out of nine patients showed mutations in TP53 (Cristofanilli and Austin, personal communication). Furthermore, TP53 mutations are more frequent in IBC (50%) than in non-inflammatory breast cancer (20 to 30%) [27],[28]. IBC is a clinical diagnosis known as the T4d category and it is a distinct clinical subtype of locally advanced breast cancer (LABC), with a particularly aggressive behavior and poor prognosis [29]. From 25 IBC patients in which NGS tissue analysis had been performed in their tumor samples, 15 patients had TP53 mutations; 5 of them had ER-positive and 10 patients ER-negative IBC; from the ER-negative IBC group, 5 patients had TNBC (Cristofanilli and Austin, personal communication).

The technologies that were used in the present work, the CellSearch™ enrichment method followed by DEPArray™ selection of CTCs, yields pure CTCs amenable for molecular characterization. Although EpCAM-based enrichment using CellSearch™ eliminates a large portion of the other circulating cells (approximately 4-log depletion), there are still considerable numbers of leukocytes present after enrichment [30]. Sieuwerts *et a*l. showed that 810 to 1,381 leukocytes remained in the sample from a total of 600 × 10^5^ leukocytes present in the 7.5 ml of blood used for the CellSearch; and it appeared that some WBCs -mainly B lymphocytes - are specifically captured by the anti-EpCAM monoclonal antibody [30]. This contamination, together with the low frequency of CTCs, creates a challenge when aiming to characterize CTCs by very sensitive molecular methods. The DEPArray™ is a new technology that allows the selection and isolation of single cells from mixed-cell populations and our results show we were able to select uncontaminated CTCs by combining the CellSearch and DEPArray™ systems. In a recent study, Peeters *et al*. [31] also used the CellSearch pre-enrichment method followed by the DEPArray system for isolatation and molecular characterization of single breast-tumor cell lines spiked in human healthy blood showing the purity of the sorted samples. However, one disadvantage of the DEPArray™ is that there is approximately 40% cell-loss [31]. One of the reasons for such loss of cells is that although the DEPArray cartridge is manually loaded with 14 μl of sample, only approximately 9.26 μl of sample is automatically injected by the system into the microchamber of the cartridge. This suggests that its applicability will likely be restricted to a limited subgroup of patients with metastatic carcinomas in whom relatively high numbers of CTCs are present in their blood.

We have recently described the existence of an EpCAM-negative epithelial-mesenchymal transition (EMT)-like subpopulation of CTCs in peripheral blood of patients with HER2-positive metastatic breast cancer [32]; therefore, it will be interesting to combine different pre-enrichment strategies with the DEPArray in order to study both EpCAM-positive and EPCAM-negative CTCs. In a recent study, Fabbri *et al*. [33] isolated single CTCs from patients with metastatic colon cancer using as a pre-enrichment method a density gradient centrifugation, Onco-Quick (Greiner BioOne), followed by DEPArray. In future studies, we will combine filtration as the pre-enrichment method with the DEPArray cell sorting.

Our studies showed the presence of different cancer sub-clones in the peripheral blood of patients with metastatic breast cancer. Cellular heterogeneity is widely reported in epithelial malignancy and it is expected that CTCs also show this condition [34]-[36]. The finding of the additional TP53 mutation in one patient, 1 year post breast biopsy, may indicate the worsening and progression of the disease. CTCs hold the key to understanding the biology of metastasis and provide a biomarker to noninvasively measure the evolution of tumor genotypes during treatment and disease progression. Our studies highlighted the importance of molecular characterization of CTCs in order to understand the biology of the disease progression.

## Conclusions

We demonstrated that by using a combination of enrichment and CTC selection methods, we are able to isolate uncontaminated CTCs to achieve single-cell molecular analysis. Our results indicate that CTCs are in fact representative of the biology of macro metastatic disease and could represent a noninvasive source of cancer cells to determine genetic markers of the disease progression and reveal the presence of potential drug targets.

## Authors’ original submitted files for images

Below are the links to the authors’ original submitted files for images. Authors’ original file for figure 1 Authors’ original file for figure 2 Authors’ original file for figure 3 Authors’ original file for figure 4 Authors’ original file for figure 5 Authors’ original file for figure 6 Authors’ original file for figure 7

## Abbreviations

AA: amino acid
APC: allophyocyanin
bp: base pairs
CK: cytokeratin
CTC: circulating tumor cell
CTM: circulating tumor microemboli
DAPI: 4’ 6 diamidino 2 phenylindole
del: deletion
EGFR: epidermal growth factor receptor
EMT: epithelial-mesenchymal transition
EpCAM: epithelial cell adhesion molecule
ER: estrogen receptor
FFPE: formalin-fixed paraffin embedded
HER2: human epidermal growth factor receptor-2
IBC: inflammatory breast cancer
LABC: locally advanced breast cancer
NGS: next-generation sequencing
PBS: phosphate-buffered saline
PE: phycoerythrin
PgR: progesterone receptor
SCLC: small-cell lung cancer
TNBC: triple-negative breast cancer
TOPOII: Type II topoisomerase
VEGF: vascular endothelial growth factor
WBC: white blood cells
WGA: whole genome amplification
