# Supplementary figures and images for: TP53 mutations detected in circulating tumor cells present in the blood of metastatic triple negative breast cancer patients
Source: Breast Cancer Res. 2014 Oct 9;16:445. doi: 10.1186/s13058-014-0445-3 (PMC4303125; doi:10.1186/s13058-014-0445-3)

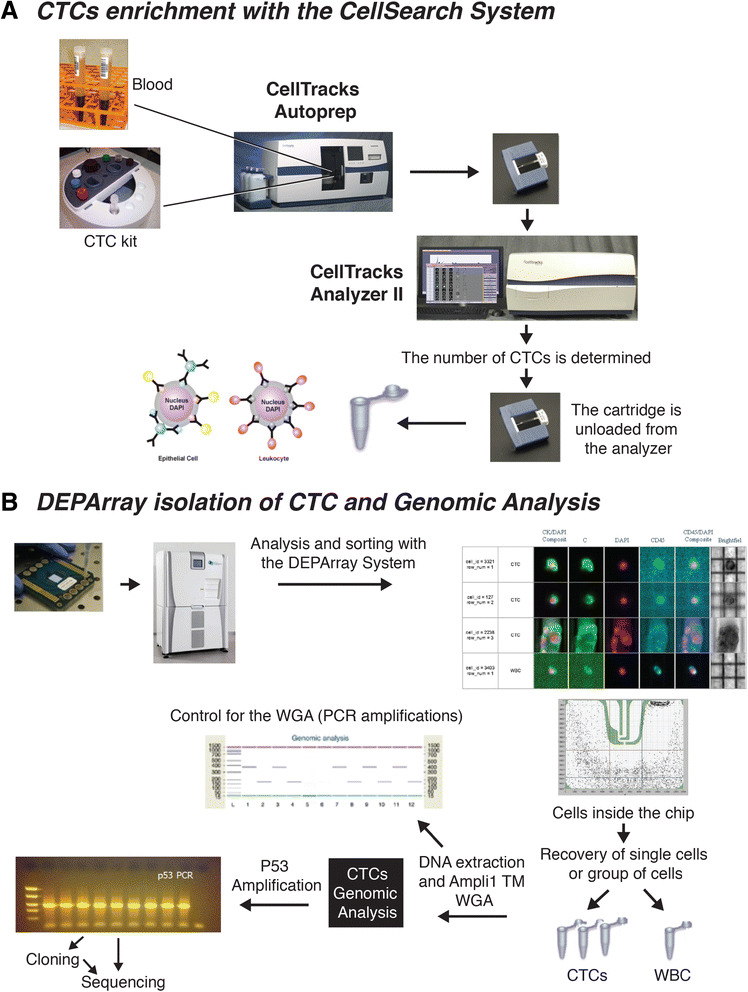

Supplement: Supplementary file 1 — Authors’ original file for figure 1 [file 13058_2014_445_MOESM1_ESM.gif]

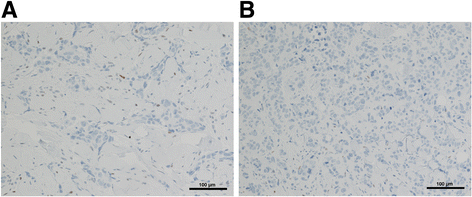

Supplement: Supplementary file 2 — Authors’ original file for figure 2 [file 13058_2014_445_MOESM2_ESM.gif]

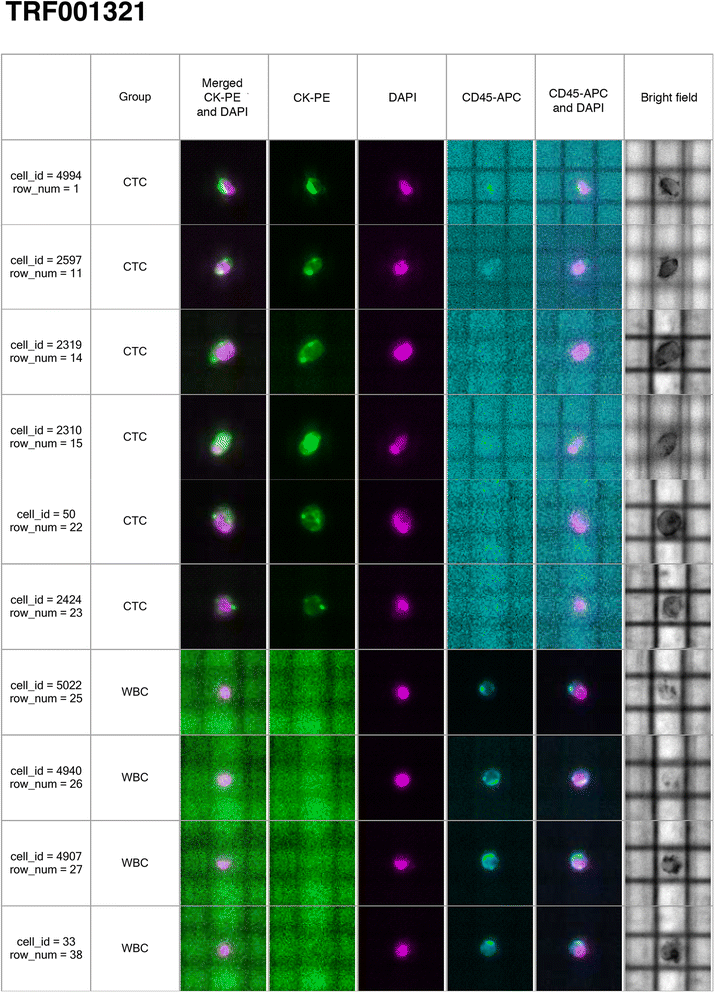

Supplement: Supplementary file 3 — Authors’ original file for figure 3 [file 13058_2014_445_MOESM3_ESM.gif]

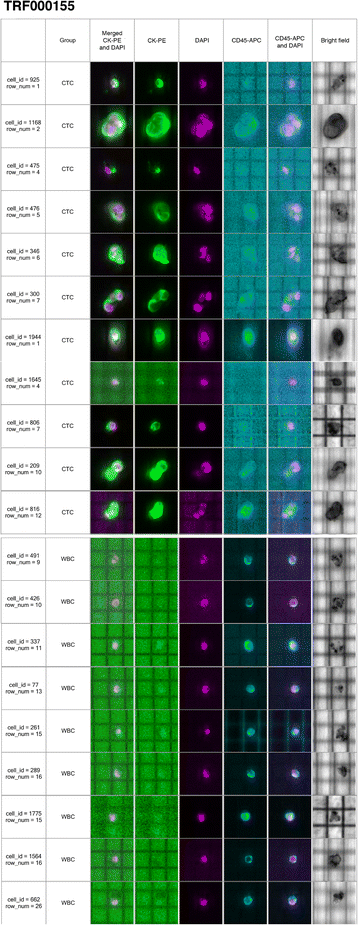

Supplement: Supplementary file 4 — Authors’ original file for figure 4 [file 13058_2014_445_MOESM4_ESM.gif]

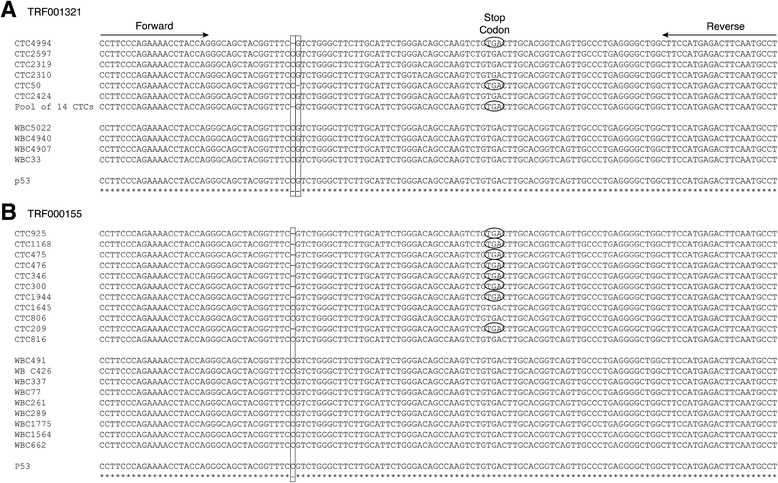

Supplement: Supplementary file 5 — Authors’ original file for figure 5 [file 13058_2014_445_MOESM5_ESM.gif]

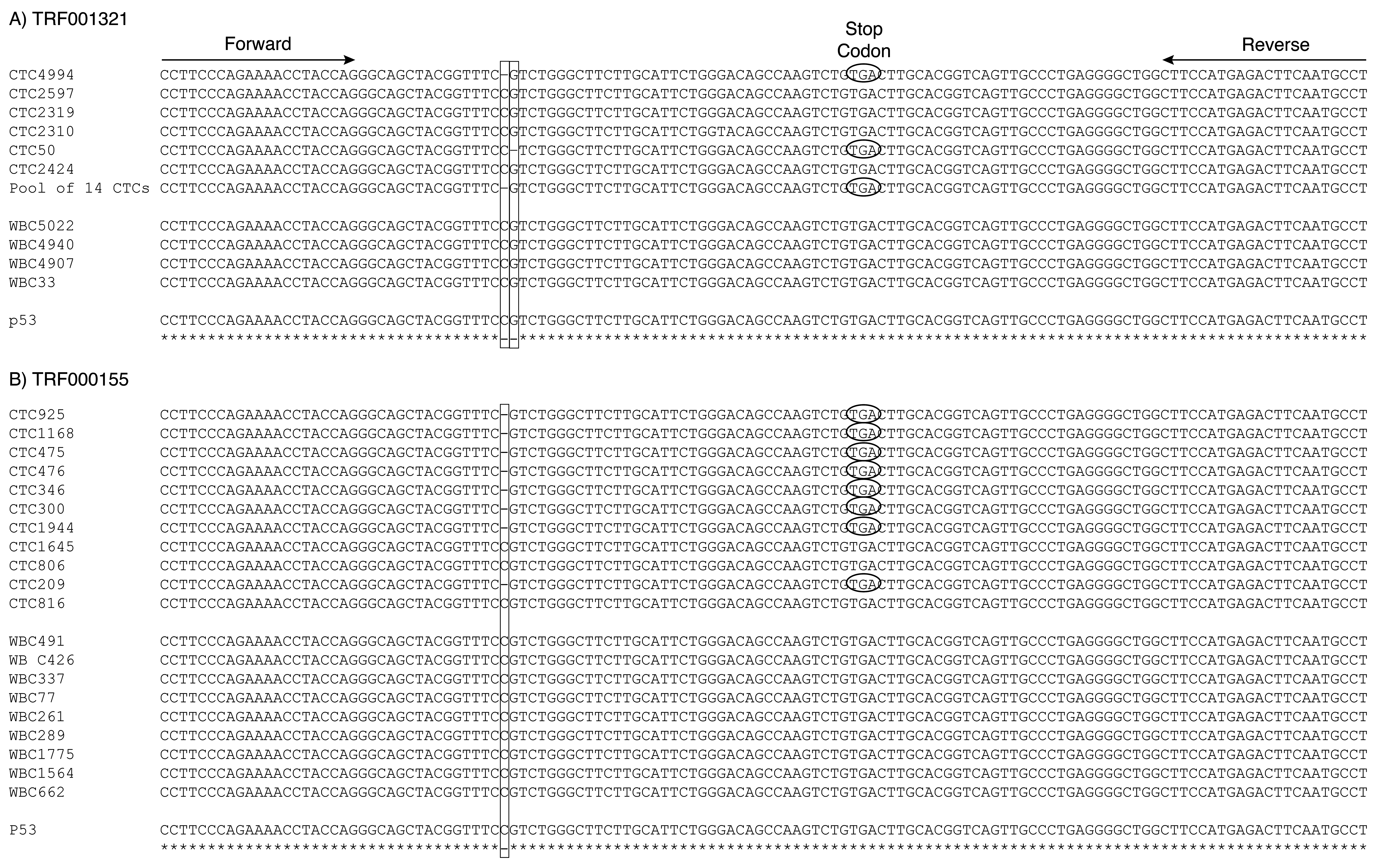

Supplement: Supplementary file 6 — Authors’ original file for figure 6 [file 13058_2014_445_MOESM6_ESM.tiff]
