# Supplementary material for: TP53 mutations detected in circulating tumor cells present in the blood of metastatic triple negative breast cancer patients
Source: Breast Cancer Res. 2014 Oct 9;16:445. doi: 10.1186/s13058-014-0445-3 (PMC4303125; doi:10.1186/s13058-014-0445-3)
Supplement: Supplementary file 7 — Authors’ original file for figure 7 [file 13058_2014_445_MOESM7_ESM.doc]

| **Patient ID** | **TP53 mutation** | |
| --- | --- | --- |
|  | **Isolated CTCs** | **Isolated WBC** |
| TRF001321 | • single CTC (4994): TP53 R110 delC fs*13  • single CTC (2597): no mutation  • single CTC (2319): no mutation  • single CTC (2310): no mutation  • single CTC (50): TP53 R110 delG fs*13  • single CTC (2424): no mutation  • pool of 14 CTCs: TP53 R110 delC fs*13 | • single WBC (5022): no mutation  • single WBC (4940): no mutation  • single WBC (4907): no mutation  • single WBC (33): no mutation |
| TRF000155 | • single CTC (925): TP53 R110 delC fs*13  • cluster of 6 CTCs (1168): TP53 R110 delC fs*13  • single CTC (475): TP53 R110 delCfs*13  • cluster of 2 CTCs (476): TP53 R110 delC fs*13  • cluster of 3 CTCs (346): TP53 R110 delC fs*13  • cluster of 3 CTCs (300): TP53 R110 delC fs*13  • single CTC (1944): TP53 R110 delC fs*13  • single CTC (1645): no mutation  • single CTC (806): no mutation  • cluster of 2 CTCs (209): TP53 R110 delC fs*13  • cluster of 3 CTCs (816): no mutation | • single WBC (491): no mutation  • single WBC (426): no mutation  • single WBC (337): no mutation  • single WBC (77): no mutation  • single WBC (261): no mutation  • single WBC (289): no mutation  • single WBC (1775): no mutation  • single WBC (1564): no mutation  • single WBC (662): no mutation |

**Table 1. CTCs harboring the TP53 exon 4 mutation and wild type allele.** CTCs and WBC were isolated from two patients with metastatic triple negative breast cancer. The cells shown in Fig. 3 and Fig. 4 were studied; cell ids are indicated (in parenthesis). As control for the wild type TP53 allele, 4 and 9 white blood cells (WBC) from patients TRF001321 and TRF000155 respectively, were isolated and TP53 were sequenced.
